# Photoautotrophic production of D-lactic acid in an engineered cyanobacterium

**DOI:** 10.1186/1475-2859-12-117

**Published:** 2013-11-25

**Authors:** Arul M Varman, Yi Yu, Le You, Yinjie J Tang

**Affiliations:** 1 Key Laboratory of Combinatory Biosynthesis and Drug Discovery (Ministry of Education), School of Pharmaceutical Sciences, Wuhan University, Wuhan 430071, China; 2 Department of Energy, Environmental and Chemical Engineering, Washington University, St. Louis, MO 63130, USA

## Abstract

**Background:**

The world faces the challenge to develop sustainable technologies to replace thousands of products that have been generated from fossil fuels. Microbial cell factories serve as promising alternatives for the production of diverse commodity chemicals and biofuels from renewable resources. For example, polylactic acid (PLA) with its biodegradable properties is a sustainable, environmentally friendly alternative to polyethylene. At present, PLA microbial production is mainly dependent on food crops such as corn and sugarcane. Moreover, optically pure isomers of lactic acid are required for the production of PLA, where D-lactic acid controls the thermochemical and physical properties of PLA. Henceforth, production of D-lactic acid through a more sustainable source (CO_2_) is desirable.

**Results:**

We have performed metabolic engineering on *Synechocystis* sp. PCC 6803 for the phototrophic synthesis of optically pure D-lactic acid from CO_2_. Synthesis of optically pure D-lactic acid was achieved by utilizing a recently discovered enzyme (i.e., a mutated glycerol dehydrogenase, GlyDH*). Significant improvements in D-lactic acid synthesis were achieved through codon optimization and by balancing the cofactor (NADH) availability through the heterologous expression of a soluble transhydrogenase. We have also discovered that addition of acetate to the cultures improved lactic acid production. More interestingly, ^13^C-pathway analysis revealed that acetate was not used for the synthesis of lactic acid, but was mainly used for synthesis of certain biomass building blocks (such as leucine and glutamate). Finally, the optimal strain was able to accumulate 1.14 g/L (photoautotrophic condition) and 2.17 g/L (phototrophic condition with acetate) of D-lactate in 24 days.

**Conclusions:**

We have demonstrated the photoautotrophic production of D-lactic acid by engineering a cyanobacterium *Synechocystis* 6803. The engineered strain shows an excellent D-lactic acid productivity from CO_2_. In the late growth phase, the lactate production rate by the engineered strain reached a maximum of ~0.19 g D-lactate/L/day (in the presence of acetate). This study serves as a good complement to the recent metabolic engineering work done on *Synechocystis* 6803 for L-lactate production. Thereby, our study may facilitate future developments in the use of cyanobacterial cell factories for the commercial production of high quality PLA.

## Background

Fossil fuels helped literally ignite the industrial revolution, and from then on radically changed the way we live; today, thousands of products are generated from fossil fuels [1]. Unfortunately, fossil fuels are non-renewable and their reserves will foreseeably run dry. Moreover, the reckless use of this resource has resulted in a tremendous release of greenhouse gases leading to adverse effects to our earth’s climate and to the creatures living on our planet. These drawbacks have driven researchers to look for alternative renewable replacements for petroleum and petroleum-derived products. Amongst the petroleum-derived products; polyethylene with an annual productivity of 80 million metric tons per annum stands out as one of the most commonly used plastics [2]. Polylactic acid (PLA) is made by the polymerization of lactic acid and has the potential to replace polyethylene as a biodegradable alternative [3]. Lactic acid is a chiral compound and exists in two isomeric forms: D (-) lactic acid and L (+) lactic acid. The various properties of polylactic acid are modulated by the mixing ratio of the D (-) and L (+) lactic acid and, henceforth, it is essential to produce both the isomers [4]. It has been estimated that for the PLA production to be profitable, the lactic acid price should be less than 0.8$/kg [5]. This necessitates the production of lactic acid from a cheaper source. Although microbial fermentation can produce lactate from sugar-based feedstock, such process may compete with global food supplies. Therefore, this work focuses on cyanobacterial process development for the sustainable synthesis of D (-) lactic acid, with CO_2_ as the carbon substrate and sunlight as an energy source.

Cyanobacteria have the ability to reduce atmospheric CO_2_ into useful organic compounds by using solar energy and have been engineered to synthesize a number of value-added products [6-9]. *Synechocystis* sp. PCC 6803 (hereafter *Synechocystis* 6803) with its ability to uptake foreign DNA naturally, has been the model organism of choice for various metabolic engineering works [10-12]. *Synechocystis* 6803 also has the ability to grow mixotrophically with glucose and acetate [13]. Therefore, along with CO_2_, its versatile carbon metabolism allows the co-utilization of cheap organic compounds for product biosynthesis. For example, acetate abundant wastewater generated from biomass hydrolysis and anaerobic digestion [14] can be potentially used for promoting cyanobacterial productivity. More importantly, there are numerous molecular biology tools for *Synechocystis* 6803, making it an attractive organism for metabolic engineering works [15,16].

*Synechocystis* 6803 has recently been engineered for the production of L-lactate (a maximal titer of 1.8 g/L and a maximal productivity of 0.15 g/L/day) [17-19]. However, engineering *Synechocystis* 6803 for the production of optically pure D-lactate synthesis is more difficult due to the lack of an efficient D-lactate dehydrogenase. Recently, a mutated glycerol dehydrogenase (GlyDH*) was discovered by Wang et al. [20] and this enzyme was found to behave as a D-lactate dehydrogenase, exhibiting an unusually high specific activity of 6.9 units per mg protein with pyruvate and NADH as substrates. This enzyme allows a *Bacillus coagulans* strain to produce 90 g/L of D-lactate. Their work served as a motivation for us to engineer *Synechocystis* 6803 through the heterologous expression of *gldA101* (encodes GlyDH*). We found that this original enzyme was able to synthesize optically pure D-lactate in *Synechocystis* 6803. To further improve cyanobacterial productivity, we employed three strategies: 1. Codon optimization of *gldA101* (Additional file 1: Figure S1); 2. Heterologous expression of a transhydrogenase; 3. Supplementing cultures with extracellular carbon sources (such as glucose, pyruvate and acetate). The final engineered strain demonstrated a high D-lactic acid productivity and titer (titer >1 g/L).

## Results and discussion

**Figure 1:**
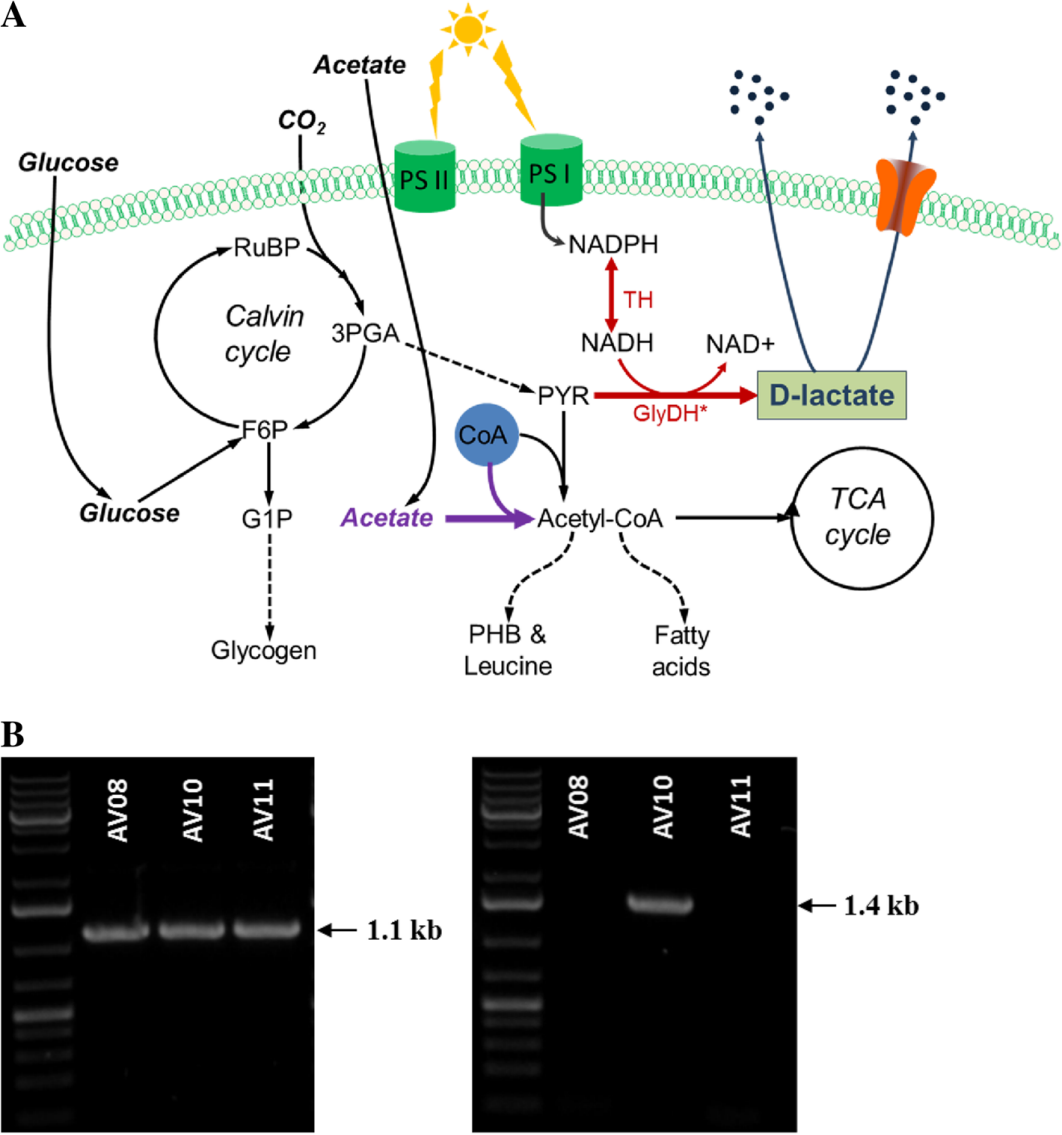
**Metabolic engineering of *Synechocystis *6803 for the synthesis of D-lactic acid. (A)** Metabolic pathway for D-lactate synthesis. Lactate permeation through the cell membrane occurs either via a lactate transporter or by passive diffusion [22,23]. Red arrows indicate the heterologous pathway engineered into *Synechocystis* 6803. **Abbreviations:** GlyDH^*^, mutant glycerol dehydrogenase; TH, Transhydrogenase; 3PGA, 3-phosphoglycerate; CoA, Coenzyme A; G1P, glucose 1-phosphate; F6P, fructose 6-phosphate; PHB, poly-β-hydroxybutyrate; RuBP, ribulose 1,5-bisphosphate. **(B)** Colony PCR to verify the presence of the heterologous genes of the mutant glycerol dehydrogenase (Left picture) and transhydrogenase (Right picture) in the engineered strains of *Synechocystis* 6803. *gldA101* was amplified with primers gldA-o-F3 and gldA-o-R; *gldA101*-*syn* was amplified with primers gldA-o-F and gldA-o-R2; *sth* was amplified with primers tranNADH-F and tranNADH-R (Table 1).

**Figure 2:**
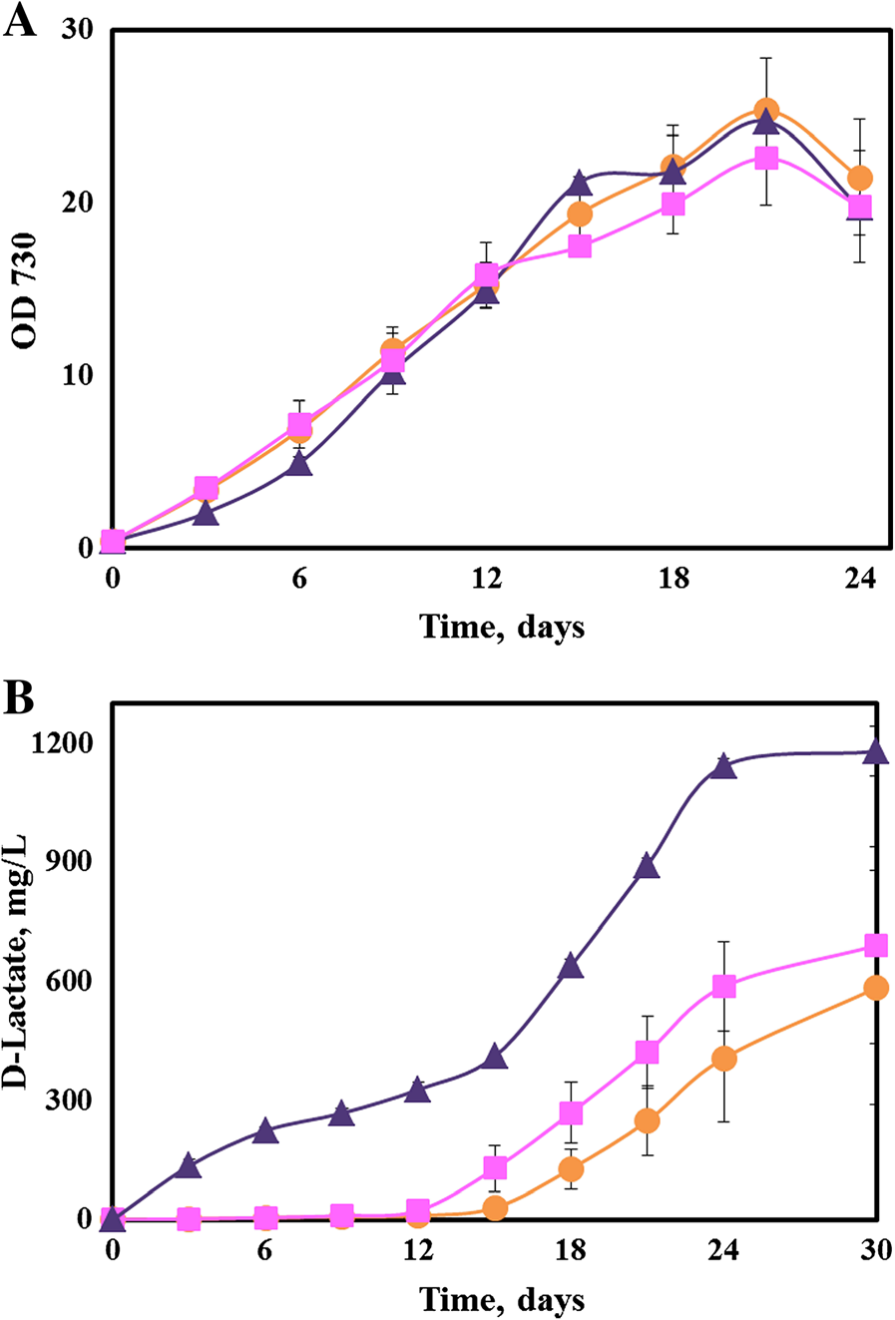
**Autotrophic production of D-Lactate in the engineered strains of *Synechocystis *6803. (A)** Growth curves and **(B)** D-lactate production in the engineered strains (n = 3). Circles: AV08 (with *gldA101*). Triangles: AV10 (with *gldA101*-*syn* and *sth*) and Squares: AV11 (with *gldA101*-*syn*).

Cyanobacteria need a lactate dehydrogenase to synthesize lactate from pyruvate (Figure 1). Earlier works on *Synechocystis* 6803 for lactate production involved the expression of an *ldh* from *Bacillus subtilis* for synthesis of L-lactate [18]. As a first step, we tested the activity of GlyDH* for D-lactate production [20] by transferring the gene from *Bacillus coagulans* to *Synechocystis* 6803. A plasmid pYY1 was constructed that contained the gene *gldA101* under the control of an Isopropyl β-D-1-thiogalactopyranoside (IPTG) inducible promoter, P_trc_. The *gldA101* gene was then subsequently transferred to the glucose tolerant wild type *Synechocystis* 6803 through natural transformation, generating the strain AV08. The optical density and the D-lactate concentration of the AV08 cultures were monitored in shake flasks. As can be verified from Figure 2, AV08 did not show any significant levels of D-lactate in the initial 12 days. The D-lactate levels started increasing steadily at the late autotrophic growth phase and reached a final titer of 0.4 g/L, whereas a wild type strain of *Synechococcus* 7002 was able to produce only ~ 7 mg/L of D-lactate through glucose fermentation [21].

A familiar strategy to increase the synthesis of a target product would be to increase the levels of the heterologous enzyme inside the cell. This can be achieved by modifying the enzyme regulation either at the transcriptional level or at the translational level. Cyanobacteria are known to have their own preference in the use of codons for synthesizing amino acids [24]. Lindberg et al. [25] have employed codon optimization for the isoprene synthase gene *IspS* and have found a 10-fold increase in the *IspS* expression level. More recently, this strategy was applied to increase the expression of the *efe* gene (from *Pseudomonas syringae*) in *Synechocystis* 6803 for ethylene production [26]. Since the gene involved in this work was borrowed from a gram-positive organism and *Synechocystis* 6803 being gram-negative, we hypothesized that this would be a useful strategy. The codon optimized gene *gldA101*-*syn* (synthesized by Genewiz Inc, South Plainfield, NJ) was integrated into the *psbA1* gene loci in the genome of the WT *Synechocystis* 6803 using the plasmid pDY3 to obtain the strain AV11.

Further improvements in product synthesis can be achieved by rectification of bottlenecks in the metabolic pathway. The lactate dehydrogenase enzyme utilizes NADH as its cofactor, whereas the ratio of NADH to NADPH is reported to be much lower in cyanobacteria. For example, the ratio of NADH to NADPH in *Synechococcus* 7942 under light conditions was estimated to be 0.15, and in *Synechocystis* 6803 under photoautotrophic conditions the intracellular NADH concentration was only 20 nmol/g fresh weight, whereas the intracellular NADPH concentration was about 140 nmol/g fresh weight [27-29]. This lower concentration of NADH in cyanobacteria, points to the fact that availability of NADH could be a major limiting factor for synthesizing D-lactate. Henceforth, a soluble transhydrogenase, *sth* from *Pseudomonas aeruginosa*[30], was introduced downstream of the gene *gldA101*-*syn.* This engineered strain was called AV10. The heterologous genes in AV10 and AV11 are under the control of the same single promoter, P_trc_, located upstream of *gldA101*-syn and *sth* in AV10 and located upstream of *gldA101*-syn in AV11.

The three strains (AV08, AV10 and AV11) showed similar growth rates to wild type strain under photoautotrophic conditions, and thus the production of D-lactate did not introduce growth defects in the engineered strains (Figure 2A and Additional file 1: Figure S2). However, the three strains differed in the production rate of D-lactic acid. The strain AV11 with codon optimization (*gldA101*-*syn*) had an improved productivity for D-lactate compared to the AV08 strain (Figure 2B). Both strains produced D-lactate mainly during the later growth stage. Introduction of the transhydrogenase improved the D-lactate synthesis further in AV10, and this strain produced D-lactate in both the growth phase and non-growth phase. The rate of photoautotrophic D-lactate production by AV10 increased significantly (achieving a maximum productivity of ~0.1 g/L/day and ~0.2 mmol/g cell/day) during the late phase of the culture and the final titer of D-lactate reached 1.14 g/L.

**Figure 3:**
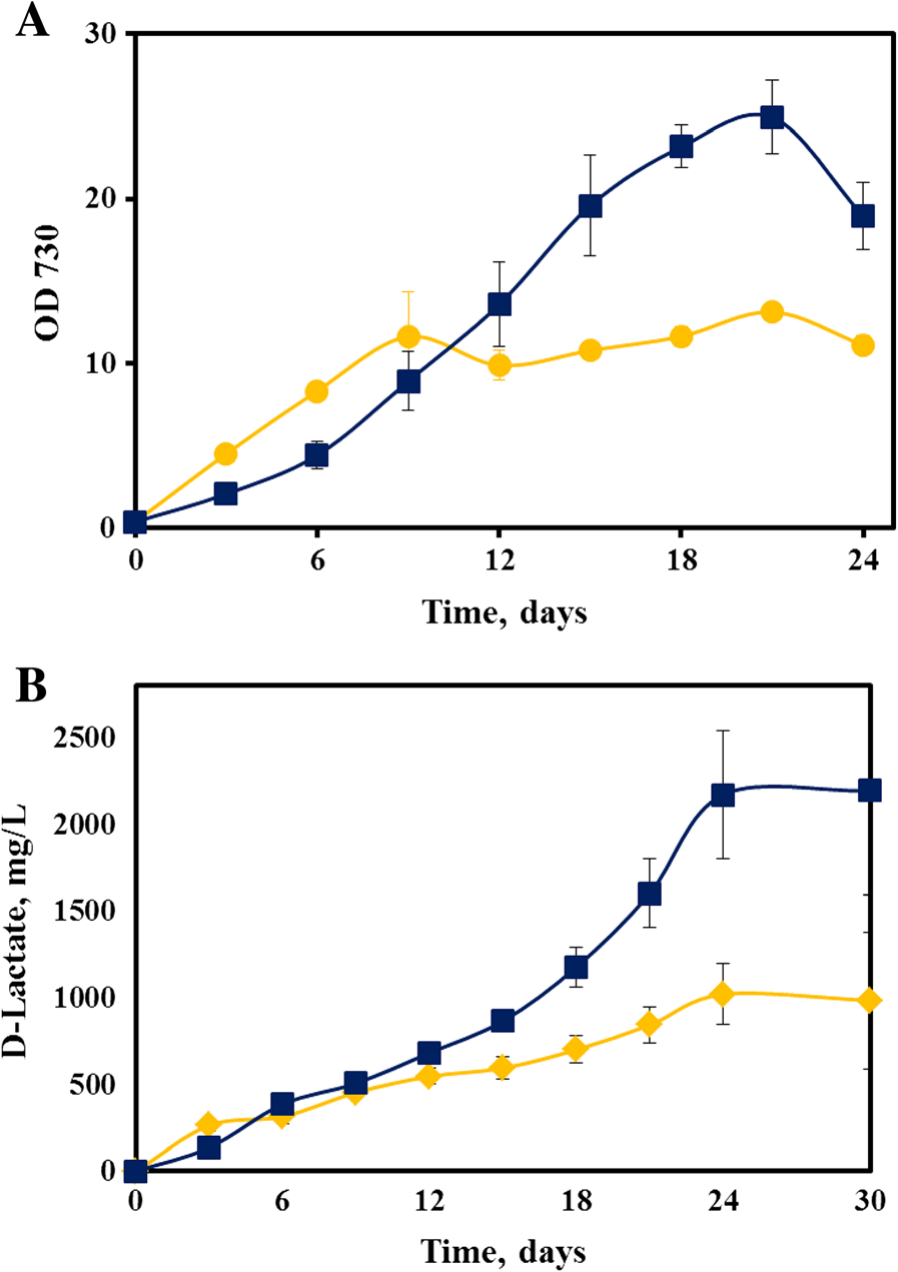
**Mixotrophic production of D-Lactate by AV10. (A)** Growth and **(B)** D-lactate production in the engineered *Synechocystis* 6803 strain AV10 (n = 3), with the provision of additional organic carbon source, i.e., with glucose and acetate (Mixotrophic metabolism). Squares: with acetate. Circles: with glucose.

We observed that the D-lactate production rate reached its peak in the later stages of cultivation, suggesting that more carbon flux has been directed to lactate production during the non-growth phase. This increased flux was expected because the lactate precursor (pyruvate) is a key metabolic node occupying a central position in the synthesis of diverse biomass components, and more pyruvate becomes available for lactate synthesis when biomass growth becomes slow. Therefore, an obvious thought would be to enhance lactate production by supplementing the cultures with pyruvate [31]. However, our experiments found that addition of pyruvate did not yield apparent improvements in D-lactate synthesis (data not shown), possibly because *Synechocystis* 6803 may lack an effective pyruvate transporter. The alternate option would be to grow AV10 with glucose and increase the glycolysis flux for pyruvate synthesis. In our previous study, addition of glucose was found to increase isobutanol production in *Synechocystis* 6803 [32]. However in this study, when we grew the AV10 strain under mixotrophic conditions (with 5 g/L glucose), it did not show a higher growth rate or display improvements in the final D-lactate titer compared to the autotrophic condition. The AV10 cultures grown in the presence of glucose instead showed an impaired growth, possibly because the engineered pathways caused a metabolic imbalance during glucose catabolism (Figure 3).

We also hypothesized that the intracellular pyruvate pool can be increased for lactate production by addition of exogenous acetate. Supplementing cultures with acetate can redirect more carbon from pyruvate to lactate in three possible ways [33]: (1) acetate is used as a building block for lactate production; (2) acetate provides additional carbon source for biomass synthesis and reduce pyruvate consumption; (3) acetate conversion by acetyl-CoA synthetase consumes Coenzyme-A (CoA), decreasing the CoA pool available for pyruvate decarboxylation. To test this hypothesis, the AV10 cultures were supplemented with 15 mM acetate. We found that growth rate of the AV10 cultures with acetate (Figure 3A) remained comparable to their growth rate under autotrophic condition, but there was substantial improvement in the synthesis of D-lactate (the maximal titer reached 2.17 g/L and the peak productivity reached ~0.19 g/L/day, Figure 3B).

**Figure 4:**
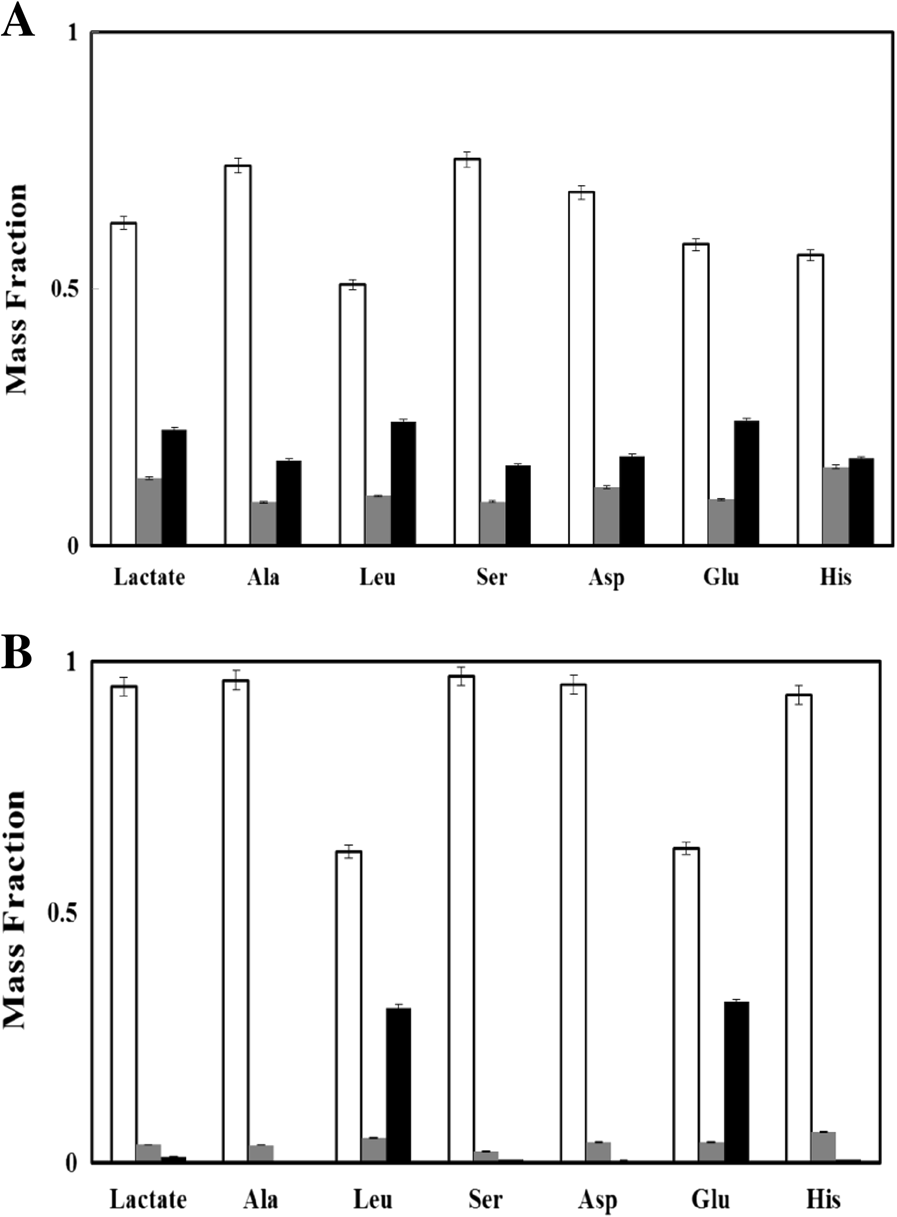
**Isotopomer analysis showing the mass fraction of isotopomers for selected proteinogenic amino acids [TBDMS based measurement] and D-lactate [MSTFA based measurement].** Standard abbreviations are used for amino acids in the figure. **(A)** Cultures grown with 5 g/L of [1,2-^13^C] glucose and **(B)** Cultures grown with 15 mM of [1,2-^13^C] acetate. "white bar" m_0_ – mass fraction without any labeled carbon; "grey bar" m_1_ – mass fraction with one labeled carbon; "black bar" m_2_ – mass fraction with two labeled carbon. (Note: natural ^13^C makes up about 1.1% of total carbon as measurement background).

To further understand the role played by glucose and acetate in D-lactate synthesis, AV10 cultures were grown with [1,2-^13^C] glucose and [1,2-^13^C] acetate (Sigma, St. Louis). Cultures were collected from the mid-log phase and were used for amino acid and D-lactate analysis. As an example, mass spectrum of D-lactate from a cyanobacterial culture is shown in Additional file 1: Figure S3. The ^13^C abundance in the amino acids and lactate were obtained as mass fraction m_i_, where 'i’ indicates the number of ^13^C in the molecule. As can be seen from Figure 4A, glucose-fed cells have significant ^13^C-carbon distributed in amino acids (indicated by an increase in m_1_ and m_2_). Also, D-lactate from glucose-fed cultures was partially ^13^C-labeled (m_2_ ~0.22). The isotopomer data in Figure 4A proved that ^13^C-glucose provided the carbon source for both biomass and lactate production. However, glucose-based mixotrophic fermentation is not beneficial to D-lactate production compared to autotrophic cultures, possibly because carbon flux from glycolysis may cause some carbon and energy imbalance [32]. As for the acetate-fed cultures, only leucine and glutamate (which both use acetyl-CoA as their precursor) were significantly labeled (an m_2_ of 0.31 and 0.32 respectively), while other amino acids (e.g., aspartate and alanine) were nonlabeled (Figure 4B). Interestingly, D-lactate from acetate-fed culture was almost nonlabeled, indicating that the carbons of lactate molecules were mainly derived from CO_2_. Therefore, the observed enhancement of lactate synthesis in the presence of acetate can be explained by two complementary mechanisms. First, acetate is an additional carbon source for synthesizing biomass building blocks, such as fatty acids and some amino acids, thus redirecting the extra carbon flux from CO_2_ to lactate. Secondly, acetate may limit the pyruvate decarboxylation reaction by reducing the CoA pool by the formation of acetyl-CoA and thus improve pyruvate availability for lactate synthesis.

## Conclusions

The results reported here are for the autotrophic production of D-lactate in cyanobacteria via the heterologous expression of a novel D-lactate dehydrogenase (GlyDH*) and by balancing the precursors and cofactors. Other molecular strategies may also be applied to further improve the D-lactate production: (1) by seeking stronger promoters [16]; (2) optimizing ribosomal binding sites [34]; (3) improving activity of GlyDH* via protein engineering; (4) introducing powerful lactate transporter [22]; (5) knocking out competing pathways (such as the glycogen and polyhydroxybutyrate synthesizing pathways); (6) duplicating the heterologous genes by integrating at multiple sites [35]; and (7) limiting biomass production by knocking down the pyruvate decarboxylation reaction. Also, considering the future outdoor algal processes for scaled up D-lactate production, we hypothesize that knocking out metabolic pathways that synthesize carbon storage molecules (polyhydroxybutyrate and glycogen) may be deleterious to algal growth during the night phase in day-night cultivation [36]. On the other hand, process optimization by employing better light conditions, along with proper CO_2_ concentration, pH and temperature control, may also be employed to increase the D-lactate productivity in a scaled-up system.

## Materials and methods

### Chemicals and reagents

Restriction enzymes, Phusion DNA polymerase, T4 DNA ligase and 10-Beta electro-competent *E. coli* kit were purchased from Fermentas or New England BioLabs. Oligonucleotides were purchased from Integrated DNA Technologies (IDT). All organic solvents, chemicals, ^13^C-labeled acetate, and glucose used in this study were purchased from Sigma-Aldrich (St. Louis, MO).

### Medium and growth conditions

*E. coli* strain 10-Beta was used as the host for all plasmids constructed in this study. *E. coli* cells were grown in liquid Luria-Bertani (LB) medium at 37°C in a shaker at 200 rpm or on solidified LB plates. Ampicillin (100 μg/mL) or kanamycin (50 μg/mL) was added to the LB medium when required for propagation of the plasmids in *E. coli*. The wild-type (glucose-tolerant) and the recombinant strain of *Synechocystis* 6803 were grown at 30°C in a liquid blue-green medium (BG-11 medium) or on solid BG-11 plates at a light intensity of 100 μmol of photons m^-2^ s^-1^ in ambient air. Kanamycin (20 μg/mL) was added to the BG-11 growth medium as required. Growth of the cells was monitored by measuring their optical density at 730 nm (OD_730_) with an Agilent Cary 60 UV–vis spectrophotometer. 10 mL cultures for the synthesis of D-lactate were grown (initial OD_730_, 0.4) in 50-mL shake flasks without any antibiotic and 1 mM Isopropyl β-D-1-thiogalactopyranoside (IPTG) was added for induction. Mixotrophic cultures of *Synechocystis* 6803 were started in BG-11 medium containing a known amount of glucose (0.5%) or acetate (15 mM) as an organic carbon source.

### Plasmid construction and transformation

The vector pTKA3 [32] served as the backbone for all the plasmids constructed in this study. The gene *gldA101* encoding GlyDH* [20], was amplified from the plasmid pQZ115 with the primers gldA-o-F2 and gldA-o-R (Tables 1 and 2). The obtained 1.2 kb fragment was digested with BamHI/NheI and cloned into the same restriction sites of pTKA3, yielding the vector pYY1. A gene cassette, which consists of the codon optimized *gldA101* (i.e., *gldA101-syn*) with the promoter P_trc_ in the upstream and the transhydrogenase (*sth*) gene from *Pseudomonas aeruginosa*[30] in the downstream, was chemically synthesized by Genewiz Inc (South Plainfield, NJ) and cloned into the commonly used *E. coli* vector pUC57-kan resulting in the plasmid vector pUC57-glda_sth. The vector pUC57-glda_sth was digested with BamHI/NheI, and the yielding 2.6 kb fragment was cloned into the corresponding restriction sites of pTKA3, resulting in the vector pDY2. The vector pDY3 was constructed by self-ligation of the 8.2 kb fragment obtained through the digestion of pDY2 with KpnI.

**Table 1:** Primer sequences

**Primer name**	**Sequence (5′ → 3′)**
gldA-o-F	GGATCCTTGACAATTAATCATCCGGCTCG
gldA-o-F2	GGATCCTTGACAATTAATCATCCGGCTCGTATAATGTGTGGAATTGTGAGCGGATAACAATTTCACACAGGAGATATAATCATATGACGAAAATCATTACCTCTCCAAGCAAGTTTATACAAGG
gldA-o-F3	ATGACGAAAATCATTACCTCTCCAAG
gldA-o-R	GCTAGCTCATGCCCATTTTTCCTTATAATACCGCCCG
gldA-o-R2	TTAGGCCCACTTTTCCTTGTAATAGC
tranNADH-F	CCTAAGCTAGCGGAGGACTAGCATGG
tranNADH-R	GCTAGCGGTACCTCAAAAAAGCCGG
ptka3-F	CCCGAAGTGGCGAGCCCGAT
CO-F	TTGATGTTGCCTTTGAACCC
O-F	ATGGATACGAAAGTGATTGC
sth-F	GAGCTACCACCTGCGCAACA
AMV17R	GCGCGACTCCCCGTCTTTGACTATCCTTTTTAGGATGGGGCA
ps1_up_fwda	TACCGGAACAGGACCAAGCCTT

**Table 2:** Plasmids and strains

**Plasmids/Strains**	**Description**	**Source or reference**
**Plasmids**		
pUC57-glda_sth	Chemically synthesized gene cassette consisting of P_trc_, *gldA101-syn* and *sth*.	Genewiz; [20,30,37]
pQZ115	Plasmid carrying *gldA101*	[20]
pTKA3	Backbone plasmid for all vectors constructed in this study, with *psbA1* as the integration loci.	[32]
pYY1	Derived from pTKA3 with *gldA101* and the promoter, P_trc_.	This study
pDY2	Derived from pTKA3 with *gldA101-syn*, *sth* and the promoter, P_trc_.	This study
pDY3	Derived from pTKA3 with *gldA101-syn* and the promoter, P_trc_.	This study
**Strains**		
*E. coli* 10-Beta	Cloning host strain.	New England Biolabs
*Synechocystis* sp. PCC 6803	Glucose tolerant wild type, naturally competent.	This study
AV08	*Synechocystis* P_trc_::*gldA101*::Km^r^, GlyDH* of *Bacillus.*	This study
AV10	*Synechocystis* P_trc_::(*gldA101-syn)-sth*::Km^r^, GlyDH* of *Bacillus,* transhydrogenase of *Pseudomonas.*	This study
AV11	*Synechocystis* P_trc_::*gldA101-syn*::Km^r^, GlyDH* of *Bacillus.*	This study

Natural transformation of *Synechocystis* 6803 was performed by using a double homologous-recombination procedure as described previously [38]. Recombinant colonies appeared between 7 and 10 days post inoculation. The genes of interest were finally integrated into the *psbA1* gene loci (a known neutral site under normal growth conditions) in the genome of *Synechocystis* 6803 [32]. For segregation, the positive colonies were propagated continuously onto BG-11 plates containing kanamycin and segregation of colonies was verified through a colony PCR with the primers AMV17R and ps1_up_fwda (Table 1). The promoter and the heterologous genes in the engineered strains were PCR amplified with respective primers (ptka3-F, CO-F, O-F, sth-F) (Table 1) and sent for sequencing to Genewiz to verify the cloning accuracy.

### D (-) lactate analysis

D(-)/L(+) lactic acid detection kit (R-biopharm) was used to measure the D-lactate concentration. Samples of the cyanobacterial culture (50 μL) were collected every 3 days and centrifuged at 12,000 rpm for 5 min. The supernatant was collected and the D-lactate concentration assay was performed following the manufacturer’s instruction. All the reactions were performed in a 96-well plate reader at room temperature (Infinite 200 PRO microplate photometer, TECAN).

### ^13^C isotopomer experiment

To estimate the carbon contributions of glucose and acetate for biomass and D-lactic acid synthesis a ^13^C labeling experiment was performed. The mutant AV10 was grown in a BG-11 medium with 0.5% glucose (1,2-^13^C_2_ glucose) or 15 mM acetate (U-^13^C_2_ acetate) (Sigma, St. Louis). Cultures were started at an OD_730_ of 0.4 and were grown with labeled glucose or acetate for over 48 hours. The biomass samples and supernatant were collected for measurement of lactate and amino acid labeling.

The proteinogenic amino acids from biomass were hydrolyzed and then derivatized with TBDMS [*N*-(tert-butyldimethylsilyl)-*N*-methyl-trifluoroacetamide], as described previously [39]. The derivatized amino acids were analyzed for their ^13^C mass fraction by GC-MS (Hewlett Packard 7890A and 5975C, Agilent Technologies, USA) equipped with a DB5-MS column (J&W Scientific) [39]. The fragment [M-57]^+^ containing information of the entire amino acid was used for calculating the ^13^C mass fractions (M: the molecular mass of the derivatized amino acids). The fragment [M-15]^+^ was used only for leucine, since its [M-57]^+^ overlaps with other mass peak [40]. To analyze extracellular D-lactic acid labeling, the supernatant (0.2 mL) was first freeze-dried at -50°C. The dried samples were then pre-derivatized with 200 μL of 2% methoxyamine hydrochloride in pyridine for 60 minutes at 37°C and then derivatized with 300 μL *N*-Methyl-*N*-(trimethylsilyl) trifluroacetamide (TMS) for 30 minutes at room temperature. The natural abundance of isotopes, including ^13^C (1.13%), ^18^O (0.20%), ^29^Si (4.70%) and ^30^Si (3.09%) changes the mass isotopomer spectrum. These changes were corrected using a published algorithm and the detailed measurement protocol can be found in our previous paper [41].

## Competing interests

The authors declare competing financial interests since this work is being covered by a pending patent application from Washington University in St. Louis.

## Authors' contributions

AMV conceived the initial idea for this research. AMV, YY, and YJT designed the experiments. AMV, YY, and YL performed the experiments. All authors read and approved the manuscript.

## Supplementary Material

Additional file 1: Figure S1 Nuleotide sequence alignment of *gldA101* and the codon-optimized *gldA101* (i.e., *gldA101-syn*, synthesized by Genewiz Inc). Conserved nucleotide sequences in *gldA101-syn* are indicated as dotted lines. **Figure S2.** Autotrophic growth curve for *Synechocystis* 6803 strains shows similar growth of the engineered D-lactate producing strains as compared to the wild type strain. Diamond: Wild type. Square: AV08. Triangle: AV10. Circle: AV11. **Figure S3.** Mass spectra obtained via GC-MS confirm the presence of lactate in the cell culture supernatant of AV10 strain. D/L lactate enzyme kit (R-Biopharm) was used to further confirm that the product is an optically pure D-lactate. Click here for file

## References

[B1] Frost JW Draths KM Knop DR Harrup MK Barker JL Niu W Chemicals from plants Carbon management: implications for R & D in the chemical sciences and technology (a workshop report to the chemical sciences roundtable) 2001 Washington, D.C: The National Academies Press 20669488

[B2] Piringer OG Baner AL Plastic packaging: interactions with food and pharmaceuticals 2008 2 Weinheim: Wiley-VCH

[B3] Vijayakumar J Aravindan R Viruthagiri T Recent trends in the production, purification and application of lactic acid Chem Biochem Eng Q 2008 22 245 264

[B4] Garlotta D A literature review of poly (lactic acid) J Polym Environ 2001 9 63 84 10.1023/A:1020200822435

[B5] Taskila S Ojamo H Kongo M The current status and future expectations in industrial production of lactic acid by lactic acid bacteria Lactic acid bacteria - R & D for food, health and livestock purposes 2013 Rijeka, Croatia: InTech

[B6] Atsumi S Higashide W Liao JC Direct photosynthetic recycling of carbon dioxide to isobutyraldehyde Nat Biotechnol 2009 27 1177 1180 10.1038/nbt.1586 19915552

[B7] Lan EI Liao JC Metabolic engineering of cyanobacteria for 1-butanol production from carbon dioxide Metab Eng 2011 13 353 363 10.1016/j.ymben.2011.04.004 21569861

[B8] Wang B Pugh S Nielsen DR Zhang W Meldrum DR Engineering cyanobacteria for photosynthetic production of 3-hydroxybutyrate directly from CO_2_ Metab Eng 2013 16 68 77 23333586 10.1016/j.ymben.2013.01.001

[B9] Kusakabe T Tatsuke T Tsuruno K Hirokawa Y Atsumi S Liao JC Hanai T Engineering a synthetic pathway in cyanobacteria for isopropanol production directly from carbon dioxide and light Metab Eng 2013 20C 101 108 24076145 10.1016/j.ymben.2013.09.007

[B10] Wang B Wang J Zhang W Meldrum DR Application of synthetic biology in cyanobacteria and algae Front Microbiol 2012 3 344 23049529 10.3389/fmicb.2012.00344 PMC3446811

[B11] Yu Y You L Liu D Hollinshead W Tang Y Zhang F Development of *Synechocystis* sp. PCC 6803 as a Phototrophic Cell Factory Mar Drugs 2013 11 2894 2916 10.3390/md11082894 23945601 PMC3766872

[B12] Berla BM Saha R Immethun CM Maranas CD Moon TS Pakrasi HB Synthetic biology of cyanobacteria: unique challenges and opportunities Front Microbiol 2013 4 246 24009604 10.3389/fmicb.2013.00246 PMC3755261

[B13] Wu GF Shen ZY Wu QY Modification of carbon partitioning to enhance PHB production in *Synechocystis* sp PCC6803 Enzyme Microb Technol 2002 30 710 715 10.1016/S0141-0229(02)00044-3

[B14] Xiao Y Ruan ZH Liu ZG Wu SG Varman AM Liu Y Tang YJJ Engineering *Escherichia coli* to convert acetic acid to free fatty acids Biochem Eng J 2013 76 60 69

[B15] Huang H-H Camsund D Lindblad P Heidorn T Design and characterization of molecular tools for a synthetic biology approach towards developing cyanobacterial biotechnology Nucleic Acids Res 2010 38 2577 2593 10.1093/nar/gkq164 20236988 PMC2860132

[B16] Huang HH Lindblad P Wide-dynamic-range promoters engineered for cyanobacteria J Biol Eng 2013 7 10 10.1186/1754-1611-7-10 23607865 PMC3724501

[B17] Joseph A Aikawa S Sasaki K Tsuge Y Matsuda F Tanaka T Kondo A Utilization of lactic acid bacterial genes in *Synechocystis* sp. PCC 6803 in the production of lactic acid Biosci Biotechnol Biochem 2013 77 966 970 10.1271/bbb.120921 23649263

[B18] Angermayr SA Paszota M Hellingwerf KJ Engineering a cyanobacterial cell factory for production of lactic acid Appl Environ Microbiol 2012 78 7098 7106 10.1128/AEM.01587-12 22865063 PMC3457509

[B19] Angermayr SA Hellingwerf KJ On the use of metabolic control analysis in the optimization of cyanobacterial biosolar cell factories J Phys Chem B 2013 117 11169 11175 10.1021/jp4013152 23506247

[B20] Wang Q Ingram LO Shanmugam KT Evolution of D-lactate dehydrogenase activity from glycerol dehydrogenase and its utility for D-lactate production from lignocellulose Proc Natl Acad Sci 2011 108 18920 18925 10.1073/pnas.1111085108 22065761 PMC3223474

[B21] McNeely K Xu Y Bennette N Bryant DA Dismukes GC Redirecting reductant flux into hydrogen production via metabolic engineering of fermentative carbon metabolism in a cyanobacterium Appl Environ Microbiol 2010 76 5032 5038 10.1128/AEM.00862-10 20543051 PMC2916493

[B22] Niederholtmeyer H Wolfstadter BT Savage DF Silver PA Way JC Engineering cyanobacteria to synthesize and export hydrophilic products Appl Environ Microbiol 2010 76 3462 3466 10.1128/AEM.00202-10 20363793 PMC2876443

[B23] Axe DD Bailey JE Transport of lactate and acetate through the energized cytoplasmic membrane of *Escherichia coli* Biotechnol Bioeng 1995 47 8 19 10.1002/bit.260470103 18623362

[B24] Campbell WH Gowri G Codon usage in higher-plants, green-algae, and cyanobacteria Plant Physiol 1990 92 1 11 10.1104/pp.92.1.1 16667228 PMC1062239

[B25] Lindberg P Park S Melis A Engineering a platform for photosynthetic isoprene production in cyanobacteria, using *Synechocystis* as the model organism Metab Eng 2010 12 70 79 10.1016/j.ymben.2009.10.001 19833224

[B26] Ungerer J Tao L Davis M Ghirardi M Maness P-C Yu J Sustained photosynthetic conversion of CO_2_ to ethylene in recombinant cyanobacterium *Synechocystis* 6803 Energy Environ Sci 2012 5 8998 9006 10.1039/c2ee22555g

[B27] Vermaas WFJ Photosynthesis and respiration in cyanobacteria 2001 In eLS: John Wiley & Sons, Ltd

[B28] Tamoi M Miyazaki T Fukamizo T Shigeoka S The Calvin cycle in cyanobacteria is regulated by CP12 via the NAD(H)/NADP(H) ratio under light/dark conditions Plant J 2005 42 504 513 10.1111/j.1365-313X.2005.02391.x 15860009

[B29] Takahashi H Uchimiya H Hihara Y Difference in metabolite levels between photoautotrophic and photomixotrophic cultures of *Synechocystis* sp. PCC 6803 examined by capillary electrophoresis electrospray ionization mass spectrometry J Exp Bot 2008 59 3009 3018 10.1093/jxb/ern157 18611912 PMC2504344

[B30] Wermuth B Kaplan NO Pyridine nucleotide transhydrogenase from *Pseudomonas aeruginosa*: purification by affinity chromatography and physicochemical properties Arch Biochem Biophys 1976 176 136 143 10.1016/0003-9861(76)90149-1 823872

[B31] Bricker TM Zhang S Laborde SM Mayer PR Frankel LK Moroney JV The malic enzyme is required for optimal photoautotrophic growth of *Synechocystis* sp. Strain PCC 6803 under continuous light but Not under a diurnal light regimen J Bacteriol 2004 186 8144 8148 10.1128/JB.186.23.8144-8148.2004 15547288 PMC529084

[B32] Varman AM Xiao Y Pakrasi HB Tang YJ Metabolic engineering of *Synechocystis* sp. Strain PCC 6803 for isobutanol production Appl Environ Microbiol 2012 79 908 914 23183979 10.1128/AEM.02827-12 PMC3568544

[B33] Wendisch VF de Graaf AA Sahm H Eikmanns BJ Quantitative determination of metabolic fluxes during coutilization of two carbon sources: comparative analyses with *Corynebacterium glutamicum* during growth on acetate and/or glucose J Bacteriol 2000 182 3088 3096 10.1128/JB.182.11.3088-3096.2000 10809686 PMC94493

[B34] Heidorn T Camsund D Huang HH Lindberg P Oliveira P Stensjo K Lindblad P Synthetic biology in cyanobacteria engineering and analyzing novel functions Methods Enzymol 2011 497 539 579 21601103 10.1016/B978-0-12-385075-1.00024-X

[B35] Gao Z Zhao H Li Z Tan X Lu X Photosynthetic production of ethanol from carbon dioxide in genetically engineered cyanobacteria Energy Environ Sci 2012 5 9857 9865 10.1039/c2ee22675h

[B36] Gründel M Scheunemann R Lockau W Zilliges Y Impaired glycogen synthesis causes metabolic overflow reactions and affects stress responses in the cyanobacterium *Synechocystis* sp. PCC 6803 Microbiology 2012 158 3032 3043 10.1099/mic.0.062950-0 23038809

[B37] Brosius J Erfle M Storella J Spacing of the -10 and -35 regions in the tac promoter. Effect on its in vivo activity J Biol Chem 1985 260 3539 3541 2579077

[B38] Zang XN Liu B Liu SM Arunakumara K Zhang XC Optimum conditions for transformation of *Synechocystis* sp. PCC 6803 J Microbiol 2007 45 241 245 17618230

[B39] You L Page L Feng X Berla B Pakrasi HB Tang YJ Metabolic pathway confirmation and discovery through ^13^C-labeling of proteinogenic amino acids J Vis Exp 2012 59 e3583 22314852 10.3791/3583 PMC3462576

[B40] Antoniewicz MR Kelleher JK Stephanopoulos G Accurate assessment of amino acid mass isotopomer distributions for metabolic flux analysis Anal Chem 2007 79 7554 7559 10.1021/ac0708893 17822305

[B41] Tang Y Shui W Myers S Feng X Bertozzi C Keasling J Central metabolism in *Mycobacterium smegmatis* during the transition from O_2_-rich to O_2_-poor conditions as studied by isotopomer-assisted metabolite analysis Biotechnol Lett 2009 31 1233 1240 10.1007/s10529-009-9991-7 19357814 PMC2709878

