# Supplementary material for: Photoautotrophic production of D-lactic acid in an engineered cyanobacterium
Source: Microb Cell Fact. 2013 Nov 25;12:117. doi: 10.1186/1475-2859-12-117 (PMC4222570; doi:10.1186/1475-2859-12-117)
Supplement: Additional file 1: Figure S1 — Nuleotide sequence alignment of gldA101 and the codon-optimized gldA101 (i.e., gldA101-syn, synthesized by Genewiz Inc). Conserved nucleotide sequences in gldA101-syn are indicated as dotted lines. Figure S2. Autotrophic growth curve for Synechocystis 6803 strains shows similar growth of the engineered D-lactate producing strains as compared to the wild type strain. Diamond: Wild type. Square: AV08. Triangle: AV10. Circle: AV11. Figure S3. Mass spectra obtained via GC-MS confirm the presence of lactate in the cell culture supernatant of AV10 strain. D/L lactate enzyme kit (R-Biopharm) was used to further confirm that the product is an optically pure D-lactate. [file 1475-2859-12-117-S1.pdf]

Supplementary materials

```
gldA101      1 ATGACGAAAATCATTACCTCTCCAAGCAAGTTTATACAAGGCCCCGATGAATTGTCCAGG
gldA-syn     1 .....C........C.....C..CTC...A..C..C..G........C..G......C..

gldA101     61 CTTTCGGCGTATACGGAAAGGCTTGGCAAAAAAGCATTTATTATTGCGGATGATTTTGTC
gldA-syn    61 T.GAGT..C.....C...C..T.G.....G..G..C.....C..C..T..C..C..C..G

gldA101    121 ACCGGCCTTGTCGGCAAAACGGTTGAAGAAAGCTATGCCGGCAAAGAAACGGGGTATCAA
gldA-syn   121 ......T.G..G..T..G..T..G..G..GTC..................C..T..C..G

gldA101    181 ATGGCATTATTCGGTGGTGAGTGTTCTAAACCGGAAATCGAACGGCTTTGTGAAATGAGC
gldA-syn   181 .....C..G..T..C..C..A..CAG...G..T..G.....G..CT.G..C..G...TC.

gldA101    241 AAATCCGAGGAAGCCGATGTCGTTGTCGGAATCGGCGGCGGAAAAACATTGGATACCGCA
gldA-syn   241 ........A........C..T..G..G..C........T..C.....C..A..C..T..C

gldA101    301 AAAGCAGTCGGGTATTACAATAACATTCCGGTGATTGTCGCGCCGACCATCGCTTCCACC
gldA-syn   301 ..G..C..G..C..C..............C.....C..G..C..C..T..T.........

gldA101    361 AATGCCCCGACAAGCGCCCTGTCTGTTATTTACAAAGAGAACGGCGAGTTTGAAGAATAC
gldA-syn   361 ........C..CTC....T..AG...G..C..T..G...........A..C..G......

gldA101    421 TTGATGCTGCCGCTGAACCCGACTTTTGTCATTATGGATACGAAAGTGATTGCCTCTGCC
gldA-syn   421 ......T....TT.......T..C.....G..C.....C..T..G.....C.....C..T

gldA101    481 CCTGCCCGCCTGCTCGTTTCCGGCATGGGAGATGCGCTTGCAACGTATTTTGAAGCGCGC
gldA-syn   481 .........T.AT.G..............C..C..CT.G..C..T..C........C...

gldA101    541 GCCACTAAGCGGGCAAATAAAACGACGATGGCAGGCGGGCGTGTTACGGAAGCGGCGATC
gldA-syn   541 ..............C.....G..C..C.....T..T..C..C.....T..G..C..C...

gldA101    601 GCGCTTGCAAAACTTTGTTATGACACGCAAATTTCGGAAGGTTTAAAAGCAAAACTGGCA
gldA-syn   601 ..CT.G..C..GT.G..C..C.....C..G..CAGT..G..C..G..G..C..GT.A..C

gldA101    661 GCGGAAAAACATCTTGTTACGGAAGCAGTGGAAAAAATCATTGAAGCGAATACGTATCTG
gldA-syn   661 ..C..G.....CT.G..G..C..G..C..T..G..G.....C..G..C..C..T..CT..

gldA101    721 AGCGGAATCGGTTCTGAAAGCGGCGGCCTTGCTGCGGCACATGCGATCCATAATGGGCTT
gldA-syn   721 TC...T..T..C..C.....T......T.A..C..C..T..C..C..T..C..C..CT.G

gldA101    781 ACCGTGCTCGAAGAAACCCATCATATGTACCACGGCGAAAAAGTGGCATTCGGTACCCTC
gldA-syn   781 ......T.G..G..G.....C..C..............G..G.....T.....C..TT.G

gldA101    841 GCCCAGCTGATTTTGGAAGATGCGCCGAAAGCGGAAATTGAAGAGGTGGTCTCCTTCTGC
gldA-syn   841 ......T....C.....G.....C..C..G..C..G.....G........G........T

gldA101    901 CTGAGTGTCGGACTTCCCGTCACGCTCGGGGATTTGGGCGTGAAAGAACTGAATGAGGAA
gldA-syn   901 T..TCC..G..CT.G..T..T..CT.G..C..C...........G...T....C......

gldA101    961 AAGCTCCGAAAAGTGGCTGAACTTTCCTGTGCGGAAGGCGAAACGATTTATAACATGCCG
gldA-syn   961 ..AT.G..G..G.....C...T.G.....C..C..G........C..C..C........C

gldA101   1021 TTTGAAGTCACGCCTGACCTTGTGTACGCAGCAATCGTTACCGCTGATTCCGTCGGGCGG
gldA-syn  1021 .....G..G..C..C..TT.G........C..C..............C.....G..C..C

gldA101   1081 TATTATAAGGAAAAATGGGCATGA
gldA-syn  1081 .....C........G.....C.A.
```

Figure S1 Nuleotide sequence alignment of *gldA101* and the codon-optimized *gldA101* (i.e., *gldA101-syn*, synthesized by Genewiz Inc). Conserved nucleotide sequences in *gldA101-syn* are indicated as dotted lines.

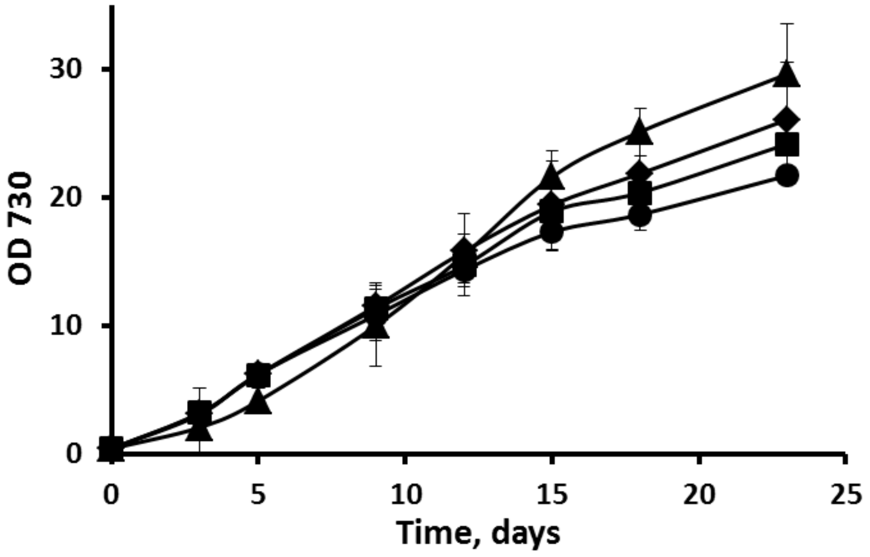

Figure S2 Autotrophic growth curve for *Synechocystis* 6803 strains shows similar growth of the engineered D-lactate producing strains as compared to the wild type strain. Diamond: Wild type. Square: AV08. Triangle: AV10. Circle: AV11.

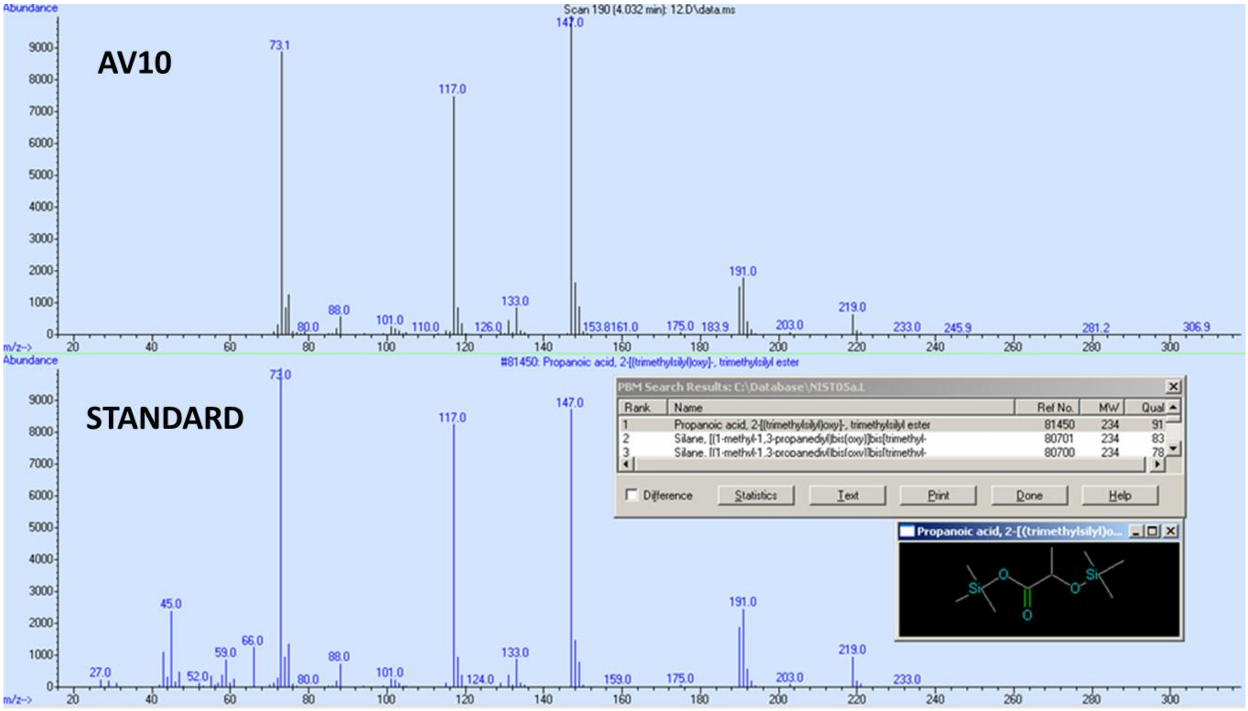

Figure S3 Mass spectra obtained via GC-MS confirm the presence of lactate in the cell culture supernatant of AV10 strain. D/L lactate enzyme kit (R-Biopharm) was used to further confirm that the product is an optically pure D-lactate.
